# Effects of Artificial Sugar Supplementation on the Composition and Nutritional Potency of Honey from *Apis cerana*

**DOI:** 10.3390/insects15050344

**Published:** 2024-05-10

**Authors:** Yueyang Hu, Jianhui Liu, Qizhong Pan, Xinxin Shi, Xiaobo Wu

**Affiliations:** 1 Honeybee Research Institute, Jiangxi Agricultural University, Nanchang 330045, China; viang97@163.com (Y.H.); liujh0507@163.com (J.L.); shixinxin0113@163.com (X.S.); 2 Jiangxi Province Key Laboratory of Honeybee Biology and Beekeeping, Nanchang 330045, China; 3 Jiangxi Anyuan Honeybee Science and Technology Backyard, Anyuan 342100, China; panqizhong1205@163.com; 4 Jiangxi Ganzhou Agricultural College, Ganzhou 341199, China

**Keywords:** artificial sugar supplementation, mature honey, physicochemical analyses, nutritional potency

## Abstract

**Simple Summary:**

In the global beekeeping industry, supplementary feeding is crucial for bee colony maintenance. Beekeepers commonly use sugar syrup as an artificial supplement, yet its impact on honey quality remains unclear. To address this gap, this study compared three types of honey: sugar-based product, honey-sourced honey, and flower-sourced honey. Flower-sourced honey exhibited superior chemical composition, antioxidant capacity, and nutritional value compared to honey-sourced honey and sugar-based product. The study emphasizes the importance of sugar source selection in shaping honey quality and highlights potential drawbacks of substituting honey with sugar syrup in traditional beekeeping practices.

**Abstract:**

In the global apiculture industry, reward feeding and supplementary feeding are essential for maintaining bee colonies. Beekeepers provide artificial supplements to their colonies, typically in the form of either a honey–water solution or sugar syrup. Owing to cost considerations associated with beekeeping, most beekeepers opt for sugar syrup. However, the effects of different types of artificial sugar supplements on bee colonies and their subsequent impact on honey composition remain unclear. To address this gap, this study compared the chemical composition, antioxidant capacity, and nutritional potency of three types of honey: honey derived from colonies fed sugar syrup (sugar-based product, SP) or a honey–water solution (honey-sourced honey, HH) and naturally sourced honey (flower-sourced honey, FH), which served as the control. The results revealed that FH outperformed HH and SP in terms of total acidity, sugar content, total protein content, and antioxidant capacity, and HH outperformed SP. Regarding nutritional efficacy, including the lifespan and learning and memory capabilities of worker bees, FH exhibited the best outcomes, with no significant differences observed between HH and SP. This study underscores the importance of sugar source selection in influencing honey quality and emphasizes the potential consequences of substituting honey with sugar syrup in traditional apiculture practices.

## 1. Introduction

Beekeeping boasts a rich history and a variety of bee-produced substances are utilized by humans. In recent times, bee products have gained significant importance in individuals’ dietary and healthcare practices [1]. Presently, bees and bee-derived products have emerged as focal points of global academic research, leading to advancements across various domains such as pollination, mutualistic symbiosis, apitherapy, and the pharmacological properties of bee products [2,3]. This underscores the significance of bees in scientific investigations concerning agricultural economics, ecosystems, and healthcare. Among the diverse array of bee products, honey stands out as the most widely utilized in daily life, finding extensive applications and serving as a traditional medicinal product [4,5].

Honey is a natural sweet substance stored by bees in bee combs through the collection and fermentation of plant nectar and honeydew [6]. It serves as a vital energy source for bees and is also a preferred sweetener for human consumption, playing a significant role in daily life and health-related preferences [7]. However, the physicochemical composition and nutritional potency of honey vary owing to factors such as the nectar source, production techniques, and beekeeping practices [8]. The quality of honey from different nectar sources can be assessed through physicochemical analysis, phenolic composition analysis, and chemometric evaluation [9]. Under natural conditions, nectar from flowers of honey-producing plants, along with honey stored in beehives, constitute the primary source of carbohydrates for bees [10].

Honey is widely recognized as a premium food product that serves as a natural food for bees [11]. During the rainy season or when honey sources are depleted, beekeepers must supplement the nutrition of the bee colony by providing sugar-based substances such as sugar syrup or honey–water [12]. However, overfeeding with sugar syrup can have detrimental effects on both the quality and composition of honey [13]. Undigested sugar syrup may be converted by bees into ‘sugar-based product’, thereby impacting the overall quality of honey within the colony [14,15]. Research had indicated that various sugar sources influence the growth and development of honey bees [16]. Honey had been found to enhance the activity of digestive enzymes in the midgut of overwintering bees, as well as promote the development of midgut tissues in these bees. Additionally, sucrose syrup and high fructose syrup had been shown to upregulate the expression of antioxidant genes in the midgut of overwintering bees [17]. The incidence of Nosema disease is higher in honey bees (*Apis mellifera*) fed with wheat starch syrup compared to those fed with honey or sugar syrup [18]. Furthermore, the composition and nutritional potency of mature honey produced from artificial sugar supplements that were not digested by worker bees, particularly the honeybee species *Apis cerana*, had not yet been studied. To address this gap, this study aimed to investigate the effects of supplementation with various sugar sources on the quality and composition of honey produced by *Apis cerana* bee colonies. To achieve this, bees were fed sugar syrup and honey–water solution, and mature honey- and sucrose-based products were obtained, referred to as sugar-based product (SP) and honey-sourced honey (HH), respectively. Additionally, flower-sourced honey (FH), obtained from undisturbed colonies, was used as a control. The nutritional potency and physicochemical composition of the two kinds of honey samples and one sample of a sugar-based product were analyzed. The results of this study would aim to compare the effects of different sugar sources provided as feed on the composition of honey and their impact on offspring bees and provide theoretical support for scientific beekeeping and the production of high-quality honey.

## 2. Materials and Methods

### 2.1. Sampling

Nine colonies of *A. cerana* with similar population sizes (approximately 10,000 bees per colony) were selected for the experiment. Prior to commencing the experiment, all honey was extracted from the honeycombs of the colonies using a standard stainless steel extraction centrifuge. Among these, three colonies were fed sugar syrup, whereas another three colonies were provided with a honey–water solution. Fresh sugar syrup and honey-water solutions with a sugar content of 50% were prepared daily and fed to the colonies. During daylight hours, the six colonies were managed within a closed system to prevent worker bees from venturing out to collect nectar. The remaining three bee colonies were left undisturbed to forage naturally for flower nectar. These colonies were bred and reared using conventional apicultural techniques between September and October 2021.

After 15 days of continuous feeding, mature honey was harvested from each colony. Initially, uncapped honey was extracted from the honeycomb using a honey extraction centrifuge. Subsequently, the honeycomb cappings from each colony were scraped off with a sterilized knife, and then honeycombs were processed in another standard honey extraction centrifuge to obtain the samples. The honey samples obtained from the sugar syrup-fed group were designated the sugar-based product (SP), and the honey from the honey–water-fed group was labeled as honey-sourced honey (HH). The honey gathered naturally from bee colonies foraging nectar was termed flower-sourced honey (FH).

In the honey production process, bee equipment such as honey extraction centrifuges, knives, and stainless-steel drums, must be cleaned, sterilized, and dried. All procedures were performed in hygienic facilities employing disinfection equipment adhering to the hygiene standards stipulated [19]. The extracted honey was stored at −20 °C prior to the experiments.

### 2.2. Methods

#### 2.2.1. Physicochemical Analysis

The moisture content and total acidity of honey were assessed according to standardized methods established by the International Honey Commission [20]. Evaluation of amylase activity in honey samples was conducted through visible and near-infrared spectroscopy [21]. The total protein content of the samples was determined utilizing the Coomassie Brilliant Blue assay [22]. The levels of glucose, fructose, sucrose, and 5-hydroxymethyl furfuraldehyde (HMF) in honey were determined using high-performance liquid chromatography (Agilent, Palo Alto, CA, USA) [23,24].

#### 2.2.2. Antioxidant Activity

##### Total Flavonoid and Total Phenolic Contents

The total flavonoid content of samples was determined using the aluminum chloride colorimetric method, and the total phenolic acid content was assessed using the Folin–Ciocalteu spectrophotometric method [25]. Honey was dissolved in ultrapure water and thoroughly mixed. Subsequently the honey–water solution was centrifuged. The supernatants of the centrifuged samples were then mixed with the respective reagents such as AlCl_3_ (XiLONG SCIENTIFIC, Shantou, China), Folin–Ciocalteu color developer (Yuanye, Shanghai, China), and Na_2_CO_3_ (XiLONG SCIENTIFIC, Shantou, China). After incubation, the mixture was transferred to a 96-well plate (Thermo Fisher Scientific, Waltham, MA, USA), and the absorbance of each sample was measured using a microplate reader (BioTek, Vermont, VT, USA). Each sample group was analyzed in quadruplicate. Standard curves were generated based on the standard solution concentrations and absorbance values to calculate the total flavonoid and phenolic acid contents of the FH, SP, and HH groups. The detailed steps followed were based on Hu’s method for determining the total flavonoid and phenolic acid content in centrifuged and pressed honey [26].

##### Antioxidant Activity Assays (Radical Scavenging Activity)

The antioxidant activities of the honey samples were evaluated using a 2,2-diphenyl-1-picrylhydrazyl (DPPH) free radical scavenging assay [27]. Additionally, the antioxidant activity of honey was assessed using a 2,2-azino-bis-3-ethylbenzothiazoline-6-sulfonic acid (ABTS) radical scavenging assay [28]. These experiments were performed utilizing the total antioxidant capacity assay kit (ABTS microplate purchased from Yuanye, Shanghai, China). The detailed steps for the free radical scavenging experiment and the calculation of the half-inhibition concentration were performed in accordance with Hu’s method [26].

#### 2.2.3. Effect of Honey with Different Sugar Sources on the Lifespan of Worker Bees

The effect of the three sugar sources on the lifespan of worker bees was assessed by the statistics of the lifespan of worker bees fed the three sugar sources. Frames with capped broods from three colonies of *A. cerana*, each with similar population sizes, were additionally selected from the same apiary as above. These frames were then transferred to a constant temperature and humidity chamber (T, 34 °C; RH, 75%, AIKANE, Shanghai, China). Worker bees newly emerged from the comb were randomly divided into four groups of around 100 bees and placed in specialized wooden boxes equipped with steel meshes on the upper and lower sides. These boxes were maintained in a chamber with constant temperature and humidity, and the worker bees were fed daily with sufficient flower-sourced honey, honey-sourced honey, sugar-based products, and sucrose solutions, with all solutions maintained at a total sugar content of 50%.

Continuous monitoring of the conditions within the specialized wooden boxes was conducted, and solutions containing various honey samples were replenished daily. Three days after the initiation of the feeding (to allow for acclimatization and excluding any potential damage to the worker bees during the transfer process), detailed records of dead worker bees are kept and then cleared daily until the entire colony perished. Subsequently, the effects of the three types of sugar sources on the lifespan of worker bees were assessed.

#### 2.2.4. Effect of Honey with Different Sugar Sources on Learning and Memory of Worker Bees

To compare the effects of foods derived from three different sugar sources on learning and memory in honeybees, a proboscis extension response (PER) conditioning experiment was performed. Worker bees newly emerged from the comb were randomly divided into four groups of approximately 60 and fed as described above. The bees were maintained for 7 d under the aforementioned controlled temperature and humidity conditions. The experiment, which included newly emerged bees from the initial three colonies, was carried out as three independent biological replicates.

In accordance with the aforementioned feeding conditions, a subset of 30 worker bees aged 7 d was selected from each group for the PER conditioning experiment. Following Hu’s experimental method, CO_2_ was used to stun the worker bees, which were then briefly placed on ice for freeze-stunning [26]. Subsequently, each worker bee was carefully immobilized within a U-shaped metal tube to ensure a secure yet not overly constrictive position. The bees were then placed in a controlled environment with regulated temperature and humidity (T, 35 °C; RH, 75%) and subjected to a 2-h period of starvation. Worker bees in poor health and those that failed to respond to sugar water were removed. After training other worker bees through positive feedback stimulation (feeding sugar water after providing lemon odor) and negative feedback stimulation (not feeding sugar water after providing vanilla odor), the memory and learning abilities of the other worker bees were assessed based on the proboscis extension response behavior when positive feedback stimulation was provided [29].

#### 2.2.5. Effect of Honey with Different Sugar Sources on the Expression of Learning- and Memory-Related Genes of Worker Bees

To assess gene expression related to learning and memory, three heads of 7-day-old worker bees were pooled together to create one sample, with each group consisting of three such composite samples [30]. The detailed steps for the extraction of total RNA and synthesis of cDNA were followed according to the protocol provided with the reagent kit (TransZol Up Plus RNA Kit, TransGen, Beijing, China; The PrimeScript™ RT Reagent Kit, Takara, Beijing, China).

*GAPDH* was selected as the internal reference gene based on learning- and memory-related genes (*AcCREB*, *Acdop2*, and *Acdop3*) in *A. cerana* [31]. These primers were synthesized by Shanghai Sangong Biotechnology Co. (Table 1).

The fluorescence quantification system and experimental procedures followed the methods outlined in the fluorescence quantification assay kit and Hu’s experimental protocol [26]. Each reaction was conducted with four technical replicates. The relative expression of each target gene was determined using the 2^−ΔΔCt^ method [32].

### 2.3. Statistical Analysis

Data analysis was conducted using StatView (version 5.0) and GraphPad Prism software (version 9.0). Mean ± standard deviation (SD) was used to express the results. And the Pearson correlation coefficient test (r) was used to assess the linear relationship between the variables. Statistical comparisons of means were performed using analysis of variance (one-way ANOVA) and Tukey’s test, with the significance level set at *p* < 0.05.

## 3. Results

### 3.1. Physicochemical Analysis

Table 2 presents the results of the study. No significant differences were observed in the moisture content or amylase activities among the SP, HH, and FH groups (*p* > 0.05). The sucrose content of the SP group was significantly higher than that of the FH and HH groups (*p* < 0.0001), with no significant difference between the FH and HH groups (*p* > 0.05). Conversely, the fructose content of the FH group was significantly higher than that of the HH and SP group (*p* < 0.0001), whereas no significant difference was observed between the HH and SP groups (*p* = 0.2258).

The total acidity content was significantly higher in the SP group than in the FH and HH groups (*p* < 0.0001), and significantly higher in the HH group compared to the FH group (*p* < 0.0001). Additionally, the glucose and total protein contents were significantly higher in the FH group than in the HH and SP groups (*p* < 0.0001), and significantly higher in the HH group compared to the SP group (*p* < 0.0001; *p* = 0.0289). Hydroxymethylfurfural (HMF) was not detected in FH, HH, or SP samples.

### 3.2. Antioxidant Activity

#### 3.2.1. Total Flavonoid and Total Phenolic Contents

**Figure 1:**
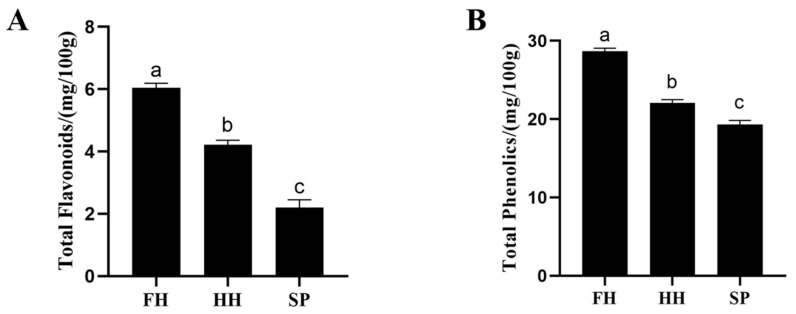
Effect of sugar source on total flavonoid content (**A**) and total phenolic content (**B**) in flower-sourced honey (FH), honey-sourced honey (HH) and sugar-based product (SP). (According to Tukey’s test, different letters in the same column denote significant differences between values (*p* < 0.05), whereas the same letters denote no significant difference (*p* > 0.05)).

The total flavonoid and phenolic contents of honey in the FH group were significantly higher than those in the HH and SP groups (*p* < 0.0001). Moreover, the HH group exhibited significantly higher levels compared to the SP group (*p* < 0.0001; *p* = 0.0016) (Figure 1A,B).

#### 3.2.2. Radical Scavenging Activity

**Figure 2:**
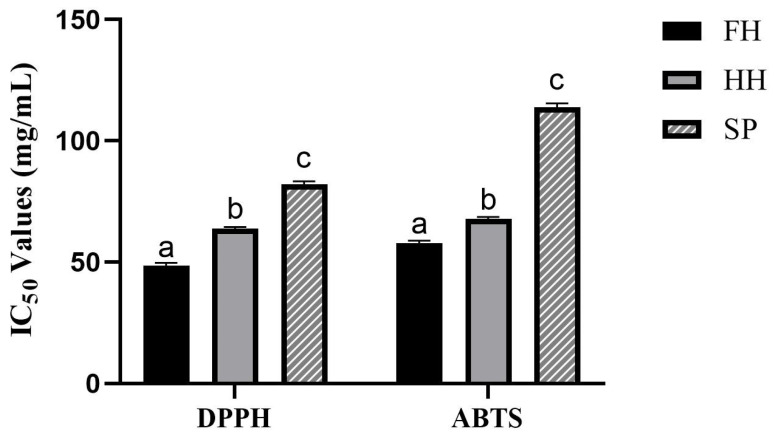
Effect of sugar source on radical scavenging activity (IC_50_-DPPH and IC_50_-ABTS assays) for the flower-sourced honey (FH), honey-sourced honey (HH), and sugar-based product (SP). (According to Tukey’s test, different letters in the same column denote significant differences between values (*p* < 0.05), whereas the same letters denote no significant difference (*p* > 0.05)).

The IC_50_ represents the concentration required to scavenge 50% of the free radicals. Lower IC_50_ values indicate a better ability to scavenge free radicals and a higher antioxidant capacity. The IC_50_-DPPH and IC_50_-ABTS values of the SP group were significantly higher than those of the FH and HH groups (*p* < 0.0001). Furthermore, the HH group demonstrated significantly higher values compared to the FH group (*p* < 0.0001) (Figure 2).

### 3.3. Lifespan Analysis

**Figure 3:**
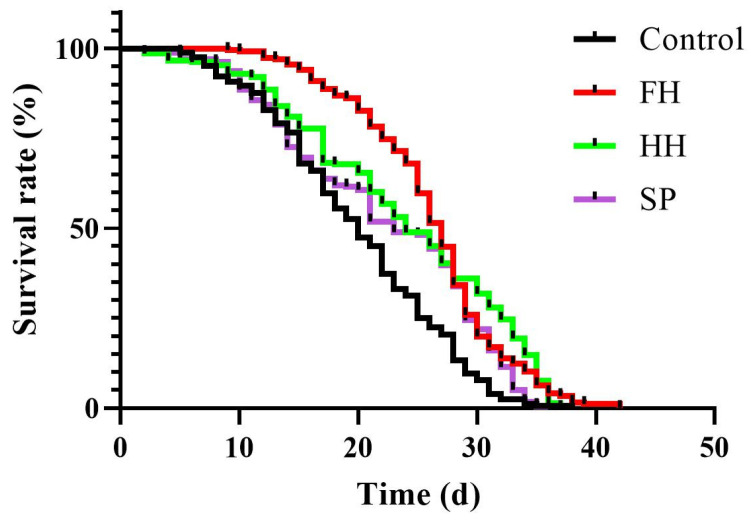
Effects of sugar-based product, honey-sourced honey, and flower-sourced honey on the survival of *A. cerana* workers.

As shown in Table 3 and Figure 3, the average lifespan of workers in the FH, HH, and SP groups was significantly *longer* than that of worker bees fed a sucrose solution (control group) (*p* < 0.0001, *p* = 0.0013, *p* = 0.0471). Additionally, the average lifespan of worker bees fed the FH diet was significantly *longer* than that of those fed the HH and SP diets (*p* = 0.0146, *p* = 0.0007).

The median lifespan of worker bees in the FH group was significantly higher than that in the HH, SP, and control groups (*p* = 0.0391, *p* = 0.0034 and *p* < 0.0001, respectively), whereas the HH group exhibited a significantly higher median lifespan compared to the control group (*p* = 0.0126). However, no significant differences in the median lifespan were observed between the HH and SP groups (*p* = 0.2247), nor between the SP and control groups (*p* = 0.0086).

### 3.4. Analysis of Memory and Learning Abilities

**Figure 4:**
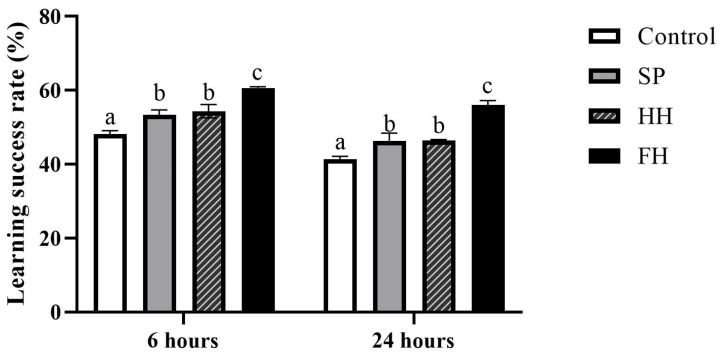
Effects of different sugar sources of honey on the learning and memory abilities of worker bees (*A. cerana*). Different letters above bars indicate significant differences between groups (ANOVA test, *p* < 0.05), whereas the same letters indicate no significant difference (*p* > 0.05).

Learning and memory abilities of worker bees at 6 h and 24 h in the FH group were significantly higher than those in the SP, HH, and control groups (*p* = 0.0031, *p* = 0.0076; *p* = 0.0004, *p* = 0.0003; *p* < 0.0001, *p* < 0.0001). Conversely, the control group exhibited significantly lower abilities compared to the SP and HH groups (*p* = 0.0173, *p* = 0.0067; *p* = 0.0158, *p* = 0.0137). Interestingly, no significant difference in learning and memory abilities was observed between the SP and HH groups at both 6 and 24 h (*p* = 0.5436, *p* = 0.9252) (Figure 4).

### 3.5. Gene Expression Analysis

**Figure 5:**
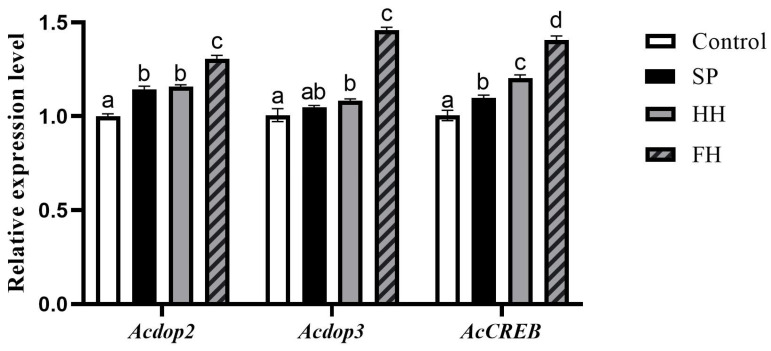
Relative expression level of genes involved in the learning and memory of worker bees and effects of different honey on the relative expression levels of worker bees. The internal control gene in each group was GAPDH. Different letters above bars indicate significant differences between groups (ANOVA test, *p* < 0.05), whereas the same letters indicate no significant difference (*p* > 0.05).

The relative expression levels of *Acdop2*, *Acdop3*, and *AcCREB* were significantly higher in the FH group compared to the HH, SP, and control groups (*p* < 0.0001). Conversely, the control group showed significantly lower expression levels compared to the HH group (*p* < 0.0001, *p* = 0.0080 and *p* = 0.0007, respectively). The relative expression of *AcCREB* was significantly higher in the HH group compared to the SP group (*p* < 0.0001), and the relative expression of *Acdop2* was significantly higher in the SP group compared to the control group (*p* < 0.0001). Notably, no significant differences in the relative expression levels of *Acdop2* and *Acdop3* were observed between the SP and HH groups (*p* = 0.4320 and *p* = 0.2040, respectively), nor in the expression of *Acdop3* between the control and SP groups (*p* = 0.1441) (Figure 5).

## 4. Discussion

Within the global apiculture industry, rewards and supplementary feeding play pivotal roles in maintaining bee colonies. Currently, sugar syrup and honey serve as the primary feed sources for bee colonies, with honey being widely regarded as the superior option [33]. However, in traditional apiculture, many beekeepers have resorted to the detrimental practice of substituting honey with sugar syrup for economic gains. This feeding method results in compromised health among bees within colonies, consequently leading to a decline in honey quality [8,34]. However, there exists a paucity of research on the effects of different sugar sources on honey quality. Therefore, this study aimed to assess the disparities in honey quality and nutritional efficacy resulting from the use of three distinct sugar sources.

Moisture content is one of the most critical indicators for evaluating honey quality, as the water content of honey indirectly influences its stability [35]. The results indicated that the moisture content of honey formed from all three sugar sources was slightly higher than the industry standards and showed no significant differences between the three groups. This is attributable to the fact that the experimental samples comprised mature honey formed through the thorough fermentation of the three sugar sources by bees. Furthermore, the moisture content of honey usually varies depending on the region of origin and the season, and is particularly high in the honey of *A. cerana* from Southern China [36]. Chemical and physical parameters such as HMF content, total acidity, and amylase value serve as important indicators of honey freshness and quality [37,38]. According to the experimental results, no HMF was detected in any of the honey samples. This is primarily because the honey samples produced in the experiment were freshly harvested and did not undergo extended storage or processing outside the hive. The amylase values of the three groups of honey samples exhibited no significant differences and exceeded industry standards, possibly because of the consistent experimental conditions, including bee species, environment, honey fermentation, and storage time, without any additional processing [39]. However, the acidity of FH was the lowest, followed by that of the HH, with SP displaying the highest acidity. This disparity may be attributed to the abundance of alkaline vitamin B present in pollen, which is stored in honey after being processed by bees, thus influencing its acidity [40]. Concurrently, the total protein content was the highest in FH, followed by HH, and lowest in SP. The primary reason for this trend may be the nutritional simplicity of sugar syrup as a feeding source, whereas flower nectar and pollen contain a rich array of nutrients, such as amino acids, proteins, vitamins, phenolics, and lipids. In contrast, honey is the product of bees freely collecting flower nectar and processing it [41].

Honey has extensive biomedical applications owing to its anti-inflammatory and antibacterial properties [42]. Research indicates that FH boasts the highest total flavonoid and phenolic acid content, along with the most potent free radical scavenging capacity, followed by HH. This suggests a hierarchy in their antioxidant capabilities: FH > HH > SP. This phenomenon is attributed to the abundance of polyphenolic compounds in the nectar consumed by bees; consequently, honey produced through nectar-processing also contains polyphenolic compounds [43].

Given their remarkable learning and memory abilities, bees can execute a range of complex social behaviors [44]. The nutritional aspects of food are crucial for the normal growth and reproduction of bees, especially affecting their lifespan and neurodevelopment [45]. In this study, bee lifespan was assessed using cage-rearing management experiments, and a PER experiment was conducted to evaluate the learning and memory abilities of bees [46]. In both the worker bee lifespan and PER experimental results, the average lifespan and learning–memory capabilities of bees in all experimental groups significantly surpassed those in the control group. The FH group exhibited the highest values, whereas the HH and SP groups showed no significant differences. This can be attributed to the fact that honey samples comprise a mixture of glucose, fructose, and sucrose, offering superior nutritional content compared to sucrose in the control group, with FH containing rich nutritional substances [47]. Furthermore, the polyphenols in honey are beneficial for brain development [48], indicating that feeding sugar syrup to bee colonies that lack adequate feed cannot fulfill the nutritional requirements of the bees. In addition, it can affect neurological development, resulting in decreased learning ability and lifespan.

To investigate the impact of feeds derived from different sugar sources on bee neurological development, experiments were conducted to assess their influence on learning- and memory-related gene expression. This study assessed the relative expression levels of three learning- and memory-related genes (*AcCREB*, *Acdop2*, and *Acdop3*) in 7-day-old worker bees fed different honey samples. Dopamine, a crucial neurotransmitter, regulates diverse behaviors and physiological processes, particularly learning and memory, by promoting CREB phosphorylation via receptor activation [49]. *AcCREB* regulates the transcription of specific genes, enhances neural cell connections, influences neuronal development, and participates in memory processes [50,51]. *Acdop2* is involved in bee motor behavior [52], while *Acdop3* is activated by a major component of the mandibular pheromone of the queen bee, high vanilla alcohol, inducing changes in cAMP levels in the brain and resulting in memory formation [53]. The relative expression of *AcCREB* in worker bees from the FH, SP, and HH groups was significantly higher than that of the control group. The FH group exhibited significantly higher levels than both the SP and HH groups, and the HH group displayed significantly higher levels than the SP group. This disparity can be attributed to the fact that FH originates from multiple nectar sources containing rich nutrients, as indicated by the aforementioned physicochemical parameters. Additionally, honey contains abundant nutrients derived from nectar following secondary metabolism. In terms of gene expression, the relative expression levels of *Acdop3* in worker bees in the FH and HH groups were significantly higher than those in worker bees in the control group. The FH group surpassed the SP and HH groups significantly; however, the SP group showed no significant difference compared with the HH and control groups. These findings may be attributed not only to differences in physicochemical parameters but also to variations in the content of vitamins B and C in honey produced from different sugar sources, thus impacting the quality and nutritional efficacy of honey [54,55]. This resulted in the following quality and efficacy hierarchy: FH > HH > SP > sucrose solution. The absence of significant differences in *Acdop2* and *Acdop3* expression between the SP and HH groups may stem from the presence of more naturally active substances from wildflower nectar in pure honey. The active compounds in honey-sourced honey may be influenced by the consumption of honey within the bee colony, affecting its proportion and efficacy. Further research and validation are necessary to confirm this hypothesis.

## 5. Conclusions

This study compared the physicochemical composition, antioxidant capacity, and nutritional efficacy of SP, HH, and FH. The results revealed that FH outperformed HH and SP in terms of total acidity, sugar content, total protein content, and antioxidant capacity. Furthermore, HH exhibited superior performance compared to SP in these aspects. When considering nutritional efficacy, including the lifespan and learning–memory capabilities of worker bees, FH demonstrated the highest values, with no significant differences observed between HH and SP.

In summary, the overall nutritional composition of honey ranked as follows: FH > HH > SP. The overall nutritional potency of honey followed the same order: FH > HH > SP > sucrose solution. Consequently, beekeepers are advised to minimize the practice of substituting honey with sugar syrup to ensure the health of bee colonies and maintain the quality of honey.

## Figures and Tables

**Table 1:** Gene primers used in the fluorescence quantitative PCR.

Gene Names	Forward Primer (5′—3′)	Reverse Primer (5′—3′)
*AcCREB*	TGAAAATCCAGTTTGATCATTCGAT	TTCAAATAATCAGCAAATCATGCAC
*Acdop2*	TTGGTTCTCCCTCTCTCCGA	CCAAGAGGTCACTATGAATGCG
*Acdop3*	AGAAGGACAAGAAAAATGCCG	CCAAGAGGTCACTATGAATGCG
*GAPDH*	GCTGGTTTCATCGATGGTTT	ACGATTTCGACCACCGTAA

**Table 2:** Results of physicochemical analysis of the flower-sourced honey, honey-sourced honey, and sugar-based product.

Process	Groups		
	Flower-Sourced Honey	Honey-Sourced Honey	Sugar-Based Product
Moisture %	21.660 ± 0.337 a	22.393 ± 0.547 a	22.213 ± 0.348 a
Amylase activity [mL/(g·h)]	27.149 ± 0.742 a	27.030 ± 0.231 a	26.868 ± 1.025 a
Total acidity (mL/kg)	24.594 ± 0.675 a	28.425 ± 0.455 b	37.829 ± 0.525 c
Fructose %	33.426 ± 0.609 a	30.427 ± 0.026 b	29.387 ± 1.748 b
Sucrose %	1.526 ± 0.044 a	1.585 ± 0.023 a	4.849 ± 0.396 b
Glucose %	43.807 ± 1.654 a	35.010 ± 0.076 b	26.986 ± 1.207 c
Total proteins (mg/100 g)	39.725 ± 3.421 a	21.571 ± 3.929 b	16.817 ± 3.124 c
HMF (mL/kg)	ND	ND	ND

According to Tukey’s test, different letters in the same column denote significant differences between values (*p* < 0.05), whereas the same letters denote no significant difference (*p* > 0.05).

**Table 3:** Effects of different types of nutrition on the average lifespan of worker bees.

Groups	Average Lifespan/Days	Median/Days	Sample Size
FH	26.743 ± 1.707 a	26.500 ± 0.500 a	236
HH	23.867 ± 0.121 b	23.500 ± 0.500 b	211
SP	22.198 ± 0.543 b	21.833 ± 1.041 bc	237
Control	20.257 ± 1.059 c	20.000 ± 2.074 c	256

According to Tukey’s test, different letters in the same column denote significant differences between values (*p* < 0.05), whereas the same letters denote no significant difference (*p* > 0.05).

## Data Availability

The raw data supporting the conclusions of this article will be made available by the authors on request.
